# Supplementary material for: Mesothelioma Mouse Models with Mixed Genomic States of Chromosome and Microsatellite Instability
Source: Cancers (Basel). 2022 Jun 24;14(13):3108. doi: 10.3390/cancers14133108 (PMC9264972; doi:10.3390/cancers14133108)
Supplement: Supplementary file 1 [file cancers-14-03108-s001.zip › cancers-1712017-supplementary.pdf]

cancers

MDPI

Supplementary Material

Mesothelioma mouse models with mixed genomic states of chromosome and microsatellite instability

Yurong Song [1*], Shaneen S. Baxter [1], Lisheng Dai [1], Chelsea Sanders [2], Sandra Burkett [3], Ryan N. Baugher [4], Stephanie D. Mellott [4], Todd B. Young [4], Heidi E. Lawhorn [4], Simone Difilippantonio [2], Baktiar Karim [5], Yuwaraj Kadariya [6], Ligia A. Pinto [1], Joseph R. Testa [6], Robert H. Shoemaker [7]

1. Supplementary Figures

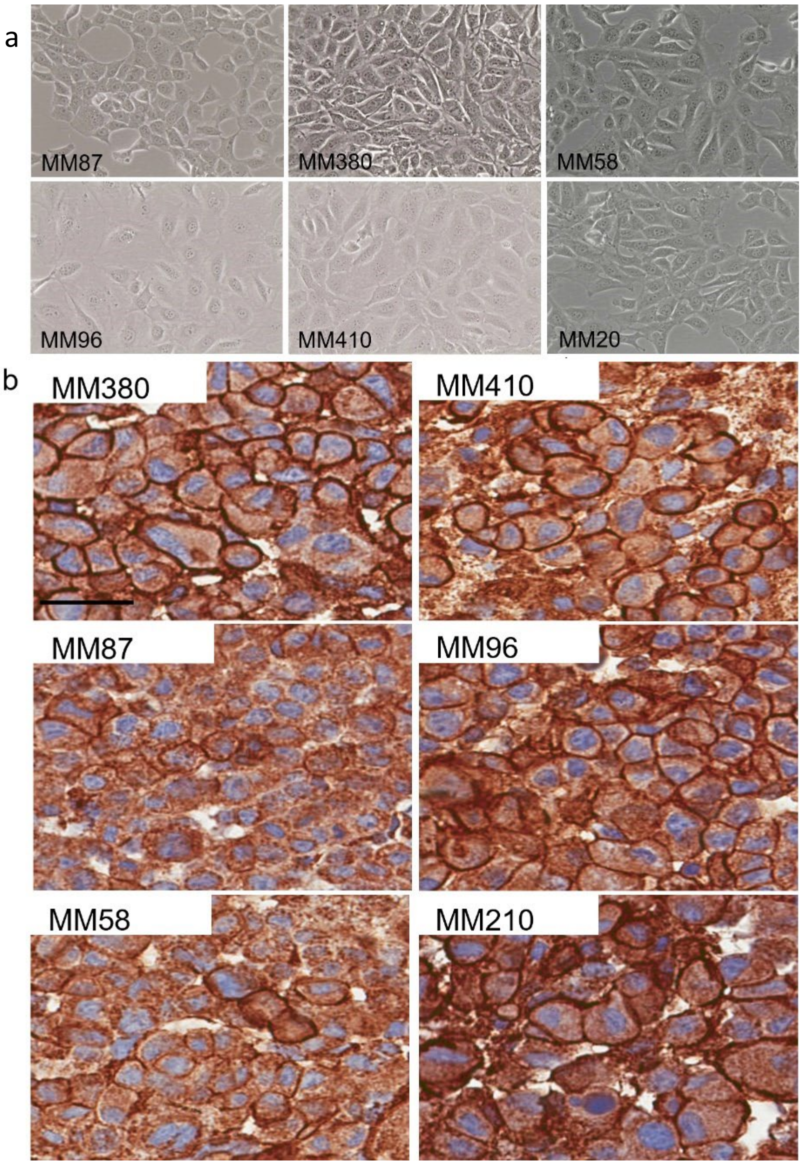

Figure S1. a. Morphology of MMe cells in culture. b. IHC staining of MSLN on FFPE sections prepared from cell pellets. Scale bar: 25um.

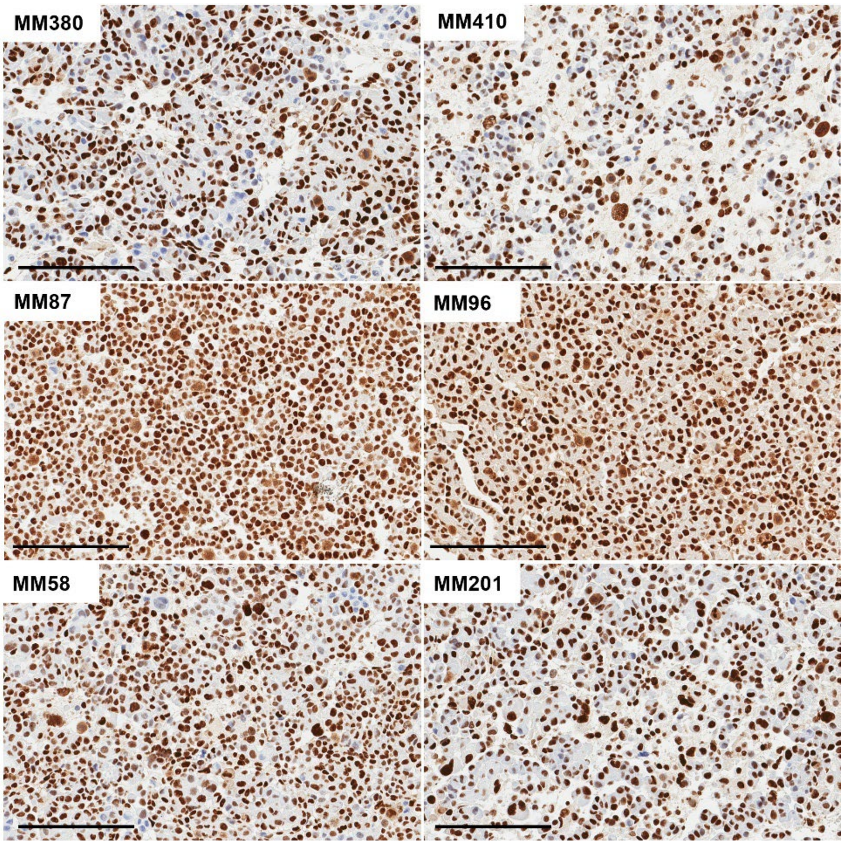

Figure S2. IHC staining of WT1 on FFPE sections prepared from cell pellets. Scale bar: 100um.

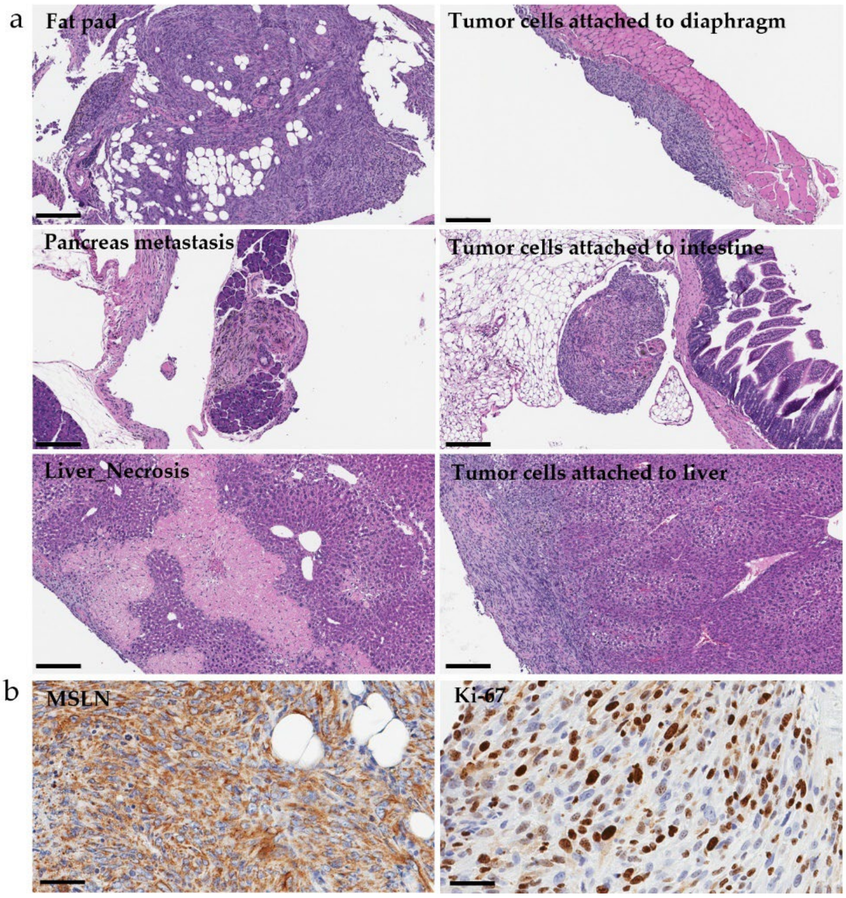

Figure S3. a. H.E. staining (scale bar: 200um) and b. IHC staining of MSLN and Ki67 (scale bar: 50um) on FFPE sections from *de novo* tumors developed in *Cdkn2a*$^{+/-}$;*Nf2*$^{+/-}$ mice exposed to asbestos.

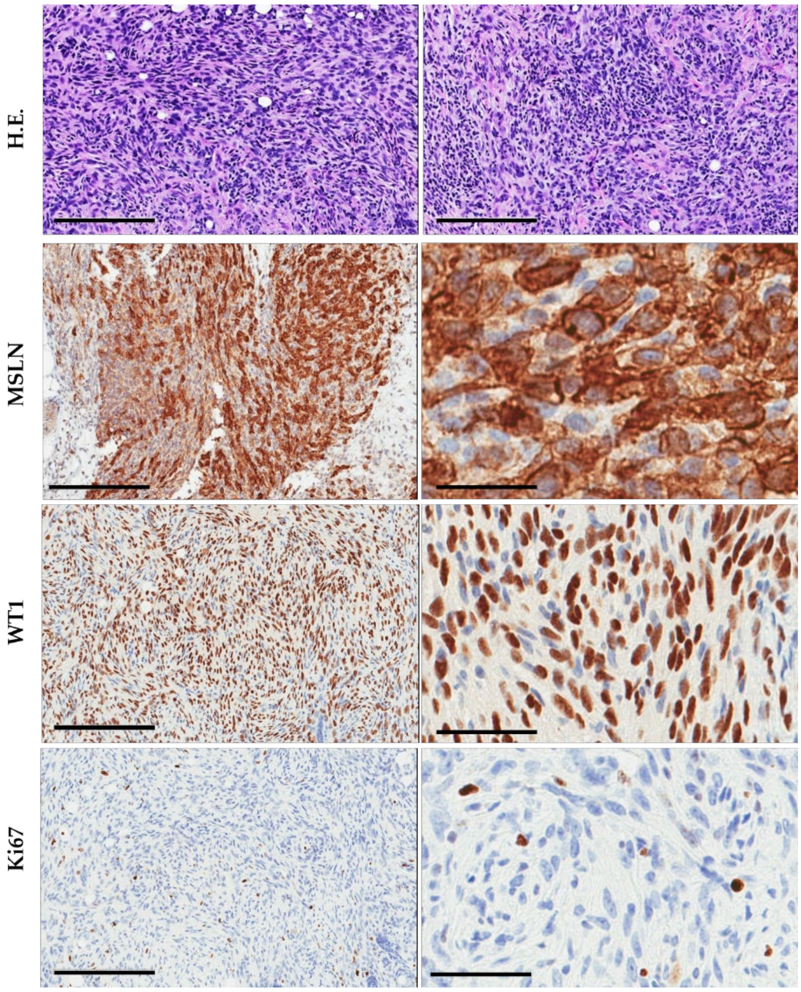

Figure S4. H.E. staining (scale bar: 200um) and IHC staining of MSLN, WT1, and Ki67 (scale bar: left panel 200um; right panel 50um) on FFPE sections of MM96 s.c. tumors.

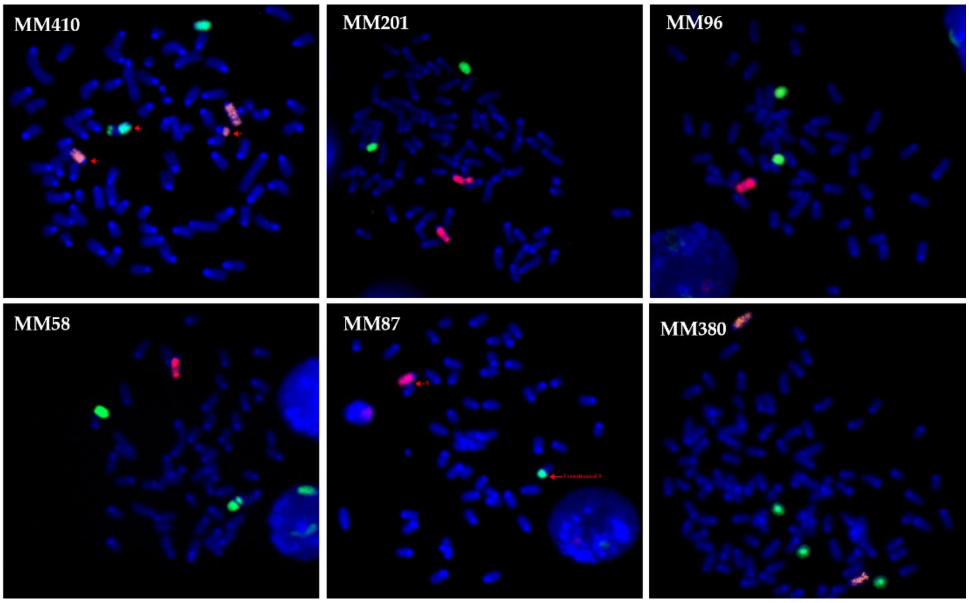

Figure S5. Chromosome X and Y specific karyotyping of six MMe cell lines. X chromosome: red/pink; Y chromosome: anti-Digoxigenin (DIG) FITC. Red arrows indicate translocations.

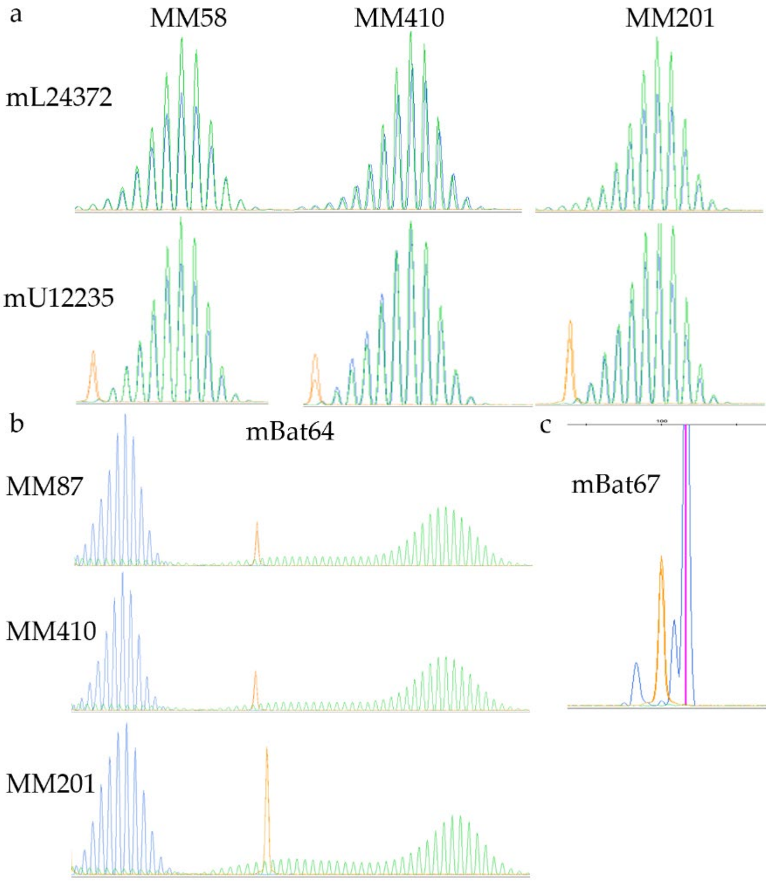

Figure S6. MSI status in plasma cfDNA from tumor-bearing animals inoculated with MMe cells. a. mL247372 and mU12235 in cfDNA from animals injected with MM58, MM410 or MM201 cells. b. mBat64 in cfDNA from animals injected with MM87, MM410 or MM201 cells. c. mBat67 in cfDNA from animals injected with MM201 cells.

2. Supplementary Tables

Table S1. Summary of antibodies used for IHC staining.

Antibody	Vendor	Catalog#	Dilution
Ki67	Millipore	AB9260	1:250
WT1	Abcam	AB89901	1:500
Mesothelin (MSLN)	IBL America	28127	1:800
TERT	Millipore	AB9260	1:250

Table S2. Primers used for MSI assessment by fragment analysis and Sanger sequencing.

Target	Fragment Analysis Forward Primer	Fragment Analysis Reverse Primer	Sanger Forward Primer	Sanger Reverse Primer
L24372	5′-FAM-GGGAAGACT GCTTAGGGAAGA-3′	5′-ATTTGGCTTTCAAGCATC CATA-3′	N/A	N/A
U12235	5′-FAM-GCTCATCTTC GTTCCCTGTC-3′	5′-CATTCGGTGGAAAGCT CTGA-3′	N/A	N/A
mBat64	5′-FAM-GCCCACACT CCTGAAAACAGTCAT-3′	5′-CCCTGGTGTGGCAACAT TAAGC-3′	N/A	N/A
mBat30	5′-VIC-ATTTGGCTT TCAA-GCATCCATA-3′	5′-GGGAAGACTGCTTAG GGAAGA-3′	N/A	N/A
mBat37	5′-NED-TCTGCCCAA AC-GTGCTTAAT-3′	5′-CCTGCCTGGGCTAA AA-TAGA-3′	N/A	N/A
mBat67	5′-FAM-TCCATCACGTT TA-TATTTTAACAGAA -3′	5′-TTGCCCATTTATC ATCTAGTTCAT-3′	5′- GTAAAACGACGGCCAGT TCCATCACGTTTA-TATTTTAACAGAA-3′	5′- GGAAACAGCTATGAC-CATG TTGCCCATTTATCATCTAG-TTCAT-3′

Table S3. PCR conditions for fragment analysis and Sanger sequencing.

Targets	PCR Conditions
mBat30 and mBat37	98 °C for 30 s, then 45 cycles of 98 °C for 10 s, 61 °C for 10 s, 72 °C for 15 s; followed by a final extension of 72 °C for 5 min, then holding at 4 °C.
mBat64, L24372, U12235	98 °C for 30 s, then 40 cycles of 98 °C for 10 s, 56 °C for 10 s, 72 °C for 15 s; followed by a final extension of 72 °C for 5 min, then holding at 4 °C.
mBat67*	98 °C for 30 s, then 40 cycles of 98 °C for 10 s, 58 °C for 10 s, 72 °C for 30 s; followed by a final extension of 72 °C for 5 min, then holding at 4 °C.

*Same PCR condition was used for mBAT67 primers for Fragment Analysis and Sanger sequencing.

Table S4. Genetic profiling using nine STR markers.

Marker name	MM410	MM380	MM87	MM96	MM58	MM201	FVB/NCrl
MCA-4-2	19.3	19.3	19.3	19.3	19.3	19.3	19.3
MCA-5-5	13, 14	14	14	14	14	13, 14	14
MCA-6-4	15.3	15.3	15.3, 16.3	15.3, 16.3	15.3, 16.3	15.3, 16.3	15.3
MCA-6-7	12, 17	12	12	12	12	12	12
MCA-9-2	15	15	15	15	15	15	15
MCA-12-1	20	20	20	20	20	20	20
MAC-15-3	20.3	20.3	20.3	20.3	20.3	20.3	20.3
MCA-18-3	17	17	17	17	17	17	17
MCA-X-1	26	27	26	26	26	26	26

The data is presented as number of repeats. A decimal point indicates microvariants (an incomplete repeat). Please note that a 1 base pair difference in allele sizes between the sample and the comparison profile represents run-to-run variability only. Sizes 2 base pairs or greater represent different allele sizes between the sample and the comparison profile. Usually if there are no more than 3 allele size differences it is considered originating from this strain (substrains variation).

Table S5. Summary of MHC class I haplotype expression in cell lines by flow cytometry analysis.

Cell line	MHC I haplotype	
	H-2Kq	H-2Dq/H-2Lq
MM410	96.6%	99.7%
MM380	99.9%	99.8%
MM87	99%	90.3%
MM96	98.3%	98.6%
MM58	97.4%	83.7%
MM201	78%	77.4%

Table S6. Summary of MSLN expression in cell lines by flow cytometry analysis and IHC staining.

Cell line	% MSLN + (among live cells) by flow	MSLN by IHC on cell pellet blocks
Negative control	0.1%	-
MM380	68.8%	+
MM410	54.6%	+
MM87	67.5%	+
MM96	94.1%	+
MM58	61.7%	+
MM201	49.8%	+

+, positive (not quantitative)

Table S7. Summary of MSLN, WT1, TERT, and Ki67 IHC data.

Animal ID (i.p. tumors)	MSLN	WT1	TERT	Ki67
01	+	+	+	+
02	+	+	+	+
03	+	+	+	+
04	+	+	+	+

+, positive (not quantitative)

Table S8. Karyotyping of six MMe cell lines via SKY analysis.

Cell Line	Karyotype
MM96	63,XYY,-X,Ts(1)+T(2;16),+Del(3),-4,Tet(5),Ts(6),+Del(6),+T(8;12)x3,Ts(9), Tet(10),Ts(11), +T(12;8;12),-12,T(12;8),+T(13;4),Ts(14),Ts(15),Tet(16),Ts(17),Tet(18),Tet(19)
MM87	58,XY,-1,T(1;15),Ts(2),Ts(3),Ts(5),Ts(6),CenDf(8)x3,+8,Ts(10),+T(11;Y),+Cen(11),+T(8;13),Ts14, +T(15;1),+Del(17)x2,Tet(19),+Cen,+Cen T(3;14)<br>48,XY,-1,T(1;15),Ts(5),Ts(6),CenDf(8)x2,+8,+T(8;13),Ts(14),-15,T(15;1),Ts(17),Ts(19),+Cenx2
MM410	77,X,-X,T(X;9),YY,Tet(1),+Del(3),-4,T(4;11),Ts(5),Tet(6),Tet(7),Tet(8),Tet(9),Pent(10),-11, +T(11;4)x2, +T(11;4),Ts(12),+Del(12),+T(3;13)x2,+T(14;5),Ts(14),Tet(15),Ts(16),Tet(1 7),Tet(18), Ts(19),+Del(19)x2
MM201	81,XXYY,Tet(1),Tet(2),Tet(3),Tet(4),+T(4;8)x2,Tet(5),Tet(6),Ts(7),Tet(8),Tet(9),Ts(10),Ts(11),Ts(12),+T(12;4),Tet(13),Tet(14),Pent(15),Tet(16),Tet(17),Tet(18),+T(18;12),M(X),M(Y)
MM380	64,XY,Ts(1),Tet(2),+Del(4)x2,Tet(5),Tet(6),Ts(8),Tet(10),Tet(11),Ts(12),Ts(13),Ts(14),Tet(15),Tet (17),Ts(18),Tet(19)<br>103,XXYY,Hex (1),Hex(2),Ts(3), +DIC (4;7),T(4;7)+DIC (4;4),+Del(4)x3,Hex(5),+Del(5),+Cen(5), Hex(6),Hex(6),Tet(9),Pent(10),Tet(11),Tet(12),+Del(12)x2,Ts(13),+Del(13),Pent(14),Pent (15), Tet(16),Pent(17),Tet(18),19x7<br>108,XXYYYY,+Del(X),Tet(1),Pent(2),Tet(3),+Del(4)x5,+Dup(4),Hex(5),Hex(6),Tet(7),(8)x6,Hex(10),Hex(11),Tet(12),Tet(13),+Del(13)x2,Pent(14),Hex(15),Tet(16),Pent(17),+Del(17), Ts(18), +CenDf(18),Pent(19)
MM58	55,XYY,+Del(3),Ts(5),-8,+CenDF(8)x2,+T(8;13),Ts(9),Ts(10),-11,Del(11),-13,T(8;13),+Del(13), T(13;12),Ts(14), Ts(15),Ts(17),Tet(18),Tet(19)<br>54,XYY,Ts(2),Ts(3),+T(3;5;3),Tet(6),-8,+CenDF(8)X2,+T(8;5),+Del(11),Ts(13),Ts(14),Ts(15), Ts(17),Ts(19)<br>48,XY,-4,Ts(5),Ts(6),+CenDF(8)x2,-8,T(8;13),-13x2,+Del(13),T(13;8),T(13;12),+Dup(14),Ts(17), Ts(19)<br>55,XY,Ts(2),Ts(3),Ts(5),Ts(6),+CenDF(8)x2,-8,T(8;13),Ts(10,+Del(11),?Dup(12),+Del(13), +Dup(14),-15,T(15;1),Tet(17),Ts(19)<br>52,XY,Ts(2),Ts(3)Tet(6),+CenDF(8),-8,T(8;5),+Del(13),+Dup(14),+Del(14),Ts(15),Ts(17),Ts(19)

Abbreviations: T, translocation; Ts, trisomy; Tet, tetrasomy; Pent, five copies; Hex, 6 copies; Del, deletion; Cen, Centromere (loss usually); CenDF, centromere deficiency; Del, deletion; Dic, dicentric; Dup, duplication (usually involving material from same chromosome so not a translocation of material from something else); M, marker chromosome too little material for definite characterization; The ()x the number, example (19)x7: chromosome 19, 7 copies.

Table S9. Inter-chromosomal translocations (T) observed in MMe cells.

Mouse chromosome	Inter-chromosomal translocation
1	T(1;15)
2	T(2;16)
3	T(3;14), T(3;13), T(3;5;3)
4	T(4;8), T(4;11), T(13;4), T(12;4), T(4;7)
5	T(8;5), T(3;5;3), T(14;5)
7	T(4;7)
8	T(4;8), T(8;5), T(8;12), T(8;13)
9	T(X;9)
11	T(11;4), T(11;Y)
12	T(12;8), T(12;4), T(18;12), T(13;12), T(12;8;12)
13	T(8:13), T(3;13), T(13;4), T(13;12), T(13;8)
14	T(14;5), T(3;14)
15	T(15;1)
16	T(2;16)
18	T(18;12)
X	T(X;9)
Y	T(11;Y)

Table S10. MSI status in MMe cell DNA and plasma cfDNA from animals injected with MMe cells.

**DNA**		**Fragment Analysis**						**Sanger Sequencing**
		U12235-A24	L24372-A27	mBat30	mBat37	mBat64	mBat67*	mBat67*
Cells	MM201	wt	wt	wt	wt	m43	m244	258 bp deletion
	MM87	wt	wt	wt	wt	m43	m244	258 bp deletion
	MM410	wt	wt	wt	wt	m43	m244	258 bp deletion
Plasma	MM201	wt	wt	n.a.	n.a.	m42	m244	n.a.
	MM58	wt	wt	n.a.	n.a.	m42	n.a.	n.a.
	MM410	wt	wt	n.a.	n.a.	m42	n.a.	n.a.
wt intestinal epithelial cells		wt	wt	wt	wt	wt	wt	n.a.
wt tail		wt	wt	wt	wt	wt	wt	wt

n.a., not analyzed; m, minus (deletion)

*The size of shifted peaks by fragment analysis was calculated by comparing the profile of MMe cells to that of wt control. However, the size of deletion by Sanger sequencing was calculated based on reference sequence. Thus, the size of deletion calculated using two different technologies was different.
